# Inclusion of KI67 significantly improves performance of the PREDICT prognostication and prediction model for early breast cancer

**DOI:** 10.1186/1471-2407-14-908

**Published:** 2014-12-03

**Authors:** Gordon C Wishart, Emad Rakha, Andrew Green, Ian Ellis, Hamid Raza Ali, Elena Provenzano, Fiona M Blows, Carlos Caldas, Paul DP Pharoah

**Affiliations:** Faculty of Health, Social Care & Education, Anglia Ruskin University, Cambridge, UK; Division of Oncology, School of Medicine, University of Nottingham, Nottingham, UK; Department of Oncology, University of Cambridge, Strangeways Research Laboratory, Worts Causeway, Cambridge, CB1 8RN UK

**Keywords:** Breast cancer, KI67, Prognostic model

## Abstract

**Background:**

PREDICT (http://www.predict.nhs.uk) is a prognostication and treatment benefit tool for early breast cancer (EBC). The aim of this study was to incorporate the prognostic effect of KI67 status in a new version (v3), and compare performance with the Predict model that includes HER2 status (v2).

**Methods:**

The validation study was based on 1,726 patients with EBC treated in Nottingham between 1989 and 1998. KI67 positivity for PREDICT is defined as >10% of tumour cells staining positive. ROC curves were constructed for Predict models with (v3) and without (v2) KI67 input. Comparison was made using the method of DeLong.

**Results:**

In 1274 ER+ patients the predicted number of events at 10 years increased from 196 for v2 to 204 for v3 compared to 221 observed. The area under the ROC curve (AUC) improved from 0.7611 to 0.7676 (p = 0.005) in ER+ patients and from 0.7546 to 0.7595 (p = 0.0008) in all 1726 patients (ER+ and ER-).

**Conclusion:**

Addition of KI67 to PREDICT has led to a statistically significant improvement in the model performance for ER+ patients and will aid clinical decision making in these patients. Further studies should determine whether other markers including gene expression profiling provide additional prognostic information to that provided by PREDICT.

## Background

Selection of appropriate patients for adjuvant chemotherapy following surgery for early breast cancer remains one of the greatest challenges for clinicians involved in the management of patients with early breast cancer. Recent debate has focused on patients with oestrogen receptor (ER) + tumours, following identification that ER+ tumours can be split into at least two specific molecular subtypes, Luminal A and Luminal B, with a marked difference in tumour characteristics and prognosis [1, 2]. Luminal A tumours in general have an excellent prognosis, and are unlikely to benefit from chemotherapy. Luminal B tumours have a worse prognosis than Luminal A tumours and can be identified by the high expression of specific proliferation-related genes such as KI67 or Aurora A kinase (AURKA). More recently additional subtypes of ER+ tumours have been identified [3]. The classifications based on gene expression can be recapitulated using immunohistochemistry (IHC) [4, 5]. While AURKA expression has been shown to be a more powerful prognosticator than KI67 [6], KI67 has been advocated as the marker of choice for measuring and monitoring tumour proliferation [7]. Furthermore, KI67 expression has been used with other IHC markers to identify the proliferative subgroup of HER2- & ER+ cases with a poor outcome [8], who may benefit from adjuvant chemotherapy.

PREDICT is an online prognostication and treatment benefit tool (http://www.predict.nhs.uk) that is based on clinico-pathological factors including tumour size, tumour grade, lymph node status, ER status, HER2 status and mode of detection. PREDICT was developed using cancer registry data on 5,694 women treated in East Anglia from 1999-2003. Breast cancer mortality models for ER positive and ER negative tumours were constructed using Cox proportional hazards, adjusted for known prognostic factors and mode of detection (symptomatic versus screen-detected) [9]. The Cox models were used to derive the baseline survivor function and the hazard ratio associated with each prognostic factor. PREDICT uses the baseline survivor function and the hazard ratio estimates (Table 1) to predict survival for a patient with a specific set of prognostic factors without adjuvant therapy and with adjuvant hormone therapy or chemotherapy assuming the relative risk reductions reported by the Early Breast Cancer Trialists Collaborative Group overview [10]. The survival estimates for an individual patient are based on the average co morbidity for women with breast cancer of a similar age. The original model (v1), which provides estimates of 5-and 10-year survival as well as absolute treatment benefits, has been validated in independent case-cohorts from the UK [9] and Canada [11]. HER2 status was subsequently added to PREDICT by incorporating an external estimate of the hazard ratio associated with HER2 positivity – i.e. an estimate from a different data set than that used to derive PREDICT v1. Following inclusion of HER2 status as an input variable, the updated Predict model (v2) provided better breast cancer specific survival estimates than Adjuvant, especially in the subset of patients with HER2 positive tumours [12].

**Table 1:** **Hazard ratio estimates for prognostic variables used by PREDICT** ^**1**^

Prognostic variable	Hazard ratio per unit increase in variable category	
(Categories)	ER+	ER-
Node status (0, 1, 2 to 4, 5 to 9,10+)	1.75	1.55
Tumour size in mm <10, 10 to 19, 20 to 29, 30 to 49, 50+)	1.43	1.44
Grade (Low, intermediate, high)	2.33	1.50
Screen detected	0.70	0.86

1 Published in Wishart et al. [9].

There appears little doubt that KI67 has great potential as a prognostic and predictive factor in early breast cancer [13], but integration into routine clinical management has to date been hampered by a failure to identify the optimal approach for its incorporation into prognostic tools [14–16]. This study was not intended to inform the current debate on finding the optimal threshold for KI67 positivity or to promote the value of KI67 as a prognostic marker. The aim of this study was to incorporate the prognostic effect of KI67 status in a new version of Predict (v3), and compare performance with the current Predict model that includes HER2 status (v2) in an independent patient cohort.

## Methods

### Prognostic effect of tumour KI67 status

An estimate for the prognostic effect of KI67 status was based on an analysis of data from the SEARCH (studies of epidemiology and risk factors in cancer heredity) study [6]. SEARCH is a large prospective population-based study of women diagnosed with breast cancer, including prevalent cases diagnosed before the age of 55 years during 1991–1996 and still alive in 1996, and incident cases consisting of women under the age of 70 years diagnosed after 1996. From the SEARCH study, KI67 was available for a total of 2,436 patients (1,835 ER positive, 601 ER negative) and immunohistochemical (IHC) expression was categorised into one of five groups (0%, 1-10%. 11-33%, 34-66%, >66%) according to an Allred proportion score. KI67 positivity, defined as >10% of tumour cells staining positive, was associated with a multi-variable adjusted hazard ratio (HR) for breast cancer specific mortality of 1.3 in patients with ER-positive tumours. KI67 was dichotomised because there was little evidence for any trend in the HR associated with increasing KI67 score. PREDICT v3 was generated by applying the HR associated with KI67 to the baseline hazards used in PREDICT v2 such that KI67-negative ER-positive tumours have a relative hazard of 0.89 and the KI67-positive ER-positive tumours have a relative hazard of 1.16. The relative hazard between KI67-positive and KI67-negative is then 1.3 with an average relative hazard of one. PREDICT v2 and PREDICT v3 are the same for ER-negative tumours as KI67 is not associated with prognosis in this sub-group.

### Validation study population

Data were available for 2,232 cases of invasive breast cancer treated in Nottingham from 1989-1998. Of these, 506 node-negative cases were excluded due to inadequate axillary node staging (<4 nodes sampled), leaving 1,726 patients (ER-, n = 452; ER+, n = 1,274) for the validation study. Data are presented in detail for the 1,274 ER positive patients.

Information obtained from the Nottingham dataset included age at diagnosis, histological grade, tumour size, number of positive lymph nodes, ER status, HER2 status, KI67 and type of adjuvant systemic therapy (none, chemotherapy, endocrine therapy, both). Mean imputation, with the missing value replaced by the mean for that variable, was used to account for missing data for tumour size, tumour grade, HER2 status and KI67 status. The number of cases with missing data for each variable is shown in Table 2. Chemotherapy regimens were considered to be first generation, as the patients were treated between 1989 and 1998.

**Table 2:** **Observed and predicted breast cancer deaths at ten years by clinical characteristics in ER positive cases**

	Number of cases	Breast cancer deaths (number)		
		Observed	PREDICT v2	PREDICT v3
Total		221	196	204
Age group				
<40	67	15	13	14
40-49	274	52	44	46
50-49	436	70	59	61
60+	497	84	79	83
Size				
<10	144	7	9	9
10-19	574	63	58	60
20-29	404	110	83	87
30-49	140	39	41	43
50+	11	2	4	4
Missing	1	0	0	0
Node status				
Negative	709	75	63	65
1+	241	48	39	41
2-4+	184	58	55	58
5-9+	37	21	19	20
10+	6	4	5	5
Missing	97	15	14	14
Grade				
1	235	18	10	10
2	528	72	62	63
3	395	127	111	119
Missing	116	4	13	13
HER2 status				
Negative	792	169	125	131
Positive	77	31	23	25
Missing	405	21	48	48

This research was carried out in compliance with the Helsinki Declaration. SEARCH is approved by the East of England - Cambridge Research Ethics Committee (02/5/42) and the Nottingham Breast Cancer study is approved by the Nottingham Research Ethics Committee 2 (REC number C2020313).

The primary endpoint was 10-year breast cancer specific survival (BCSS). Predicted survival was calculated for each patient using v2 and v3 of PREDICT. Model calibration was analysed as a comparison of the predicted mortality estimates from each model with the observed mortality. In addition to comparing calibration in the complete data set, we evaluated calibration within strata of other prognostic variables. We also evaluated calibration within quintile of predicted mortality. A goodness-of-fit test was carried out by using a χ^2^-test based on the observed and predicted number of events within each quintile (5 d.f.). Model discrimination was evaluated by calculating the area under the receiver-operator-characteristic curve (AUC) calculated for 10-year mortality. This is a measure of how well each version of the model identifies those patients with worse survival. The AUC is the probability that the predicted mortality from a randomly selected patient who died will be higher than the predicted mortality from a randomly selected survivor. Comparison between v2 and v3 was made using the method of DeLong [16].

## Results

### Calibration

**Figure 1:**
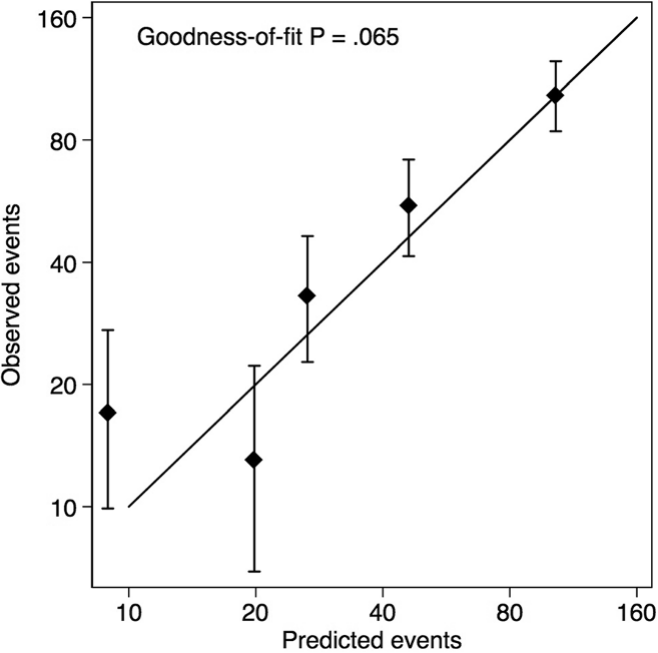
**Calibration plots of observed outcomes with 95% confidence intervals against predicted outcomes by quartiles of the predicted value.**

In the 1,274 patients with ER-positive tumours, there were 221 breast cancer deaths after ten years of follow-up. The calibration of PREDICT v2 and PREDICTv3 was good with PREDICT v3 slightly out performing v2. V2 of PREDICT estimated 196 deaths compared to 204 deaths estimated by v3. The observed and predicted numbers of deaths by clinical characteristics are shown in Table 2. PREDICT performed well in all sub groups, with v3 performing better than v2 in all but the cases with large tumours (>30 mm) or cases with ten or more positive nodes. Calibration of PREDICT v3 across quintiles of predicted risk was good (Figure 1, goodness-of-fit P = .065). The number of deaths in the 453 ER-negative cases predicted by PREDICT v2/v3 was the same as the number observed (n = 142).

### Discrimination

**Figure 2:**
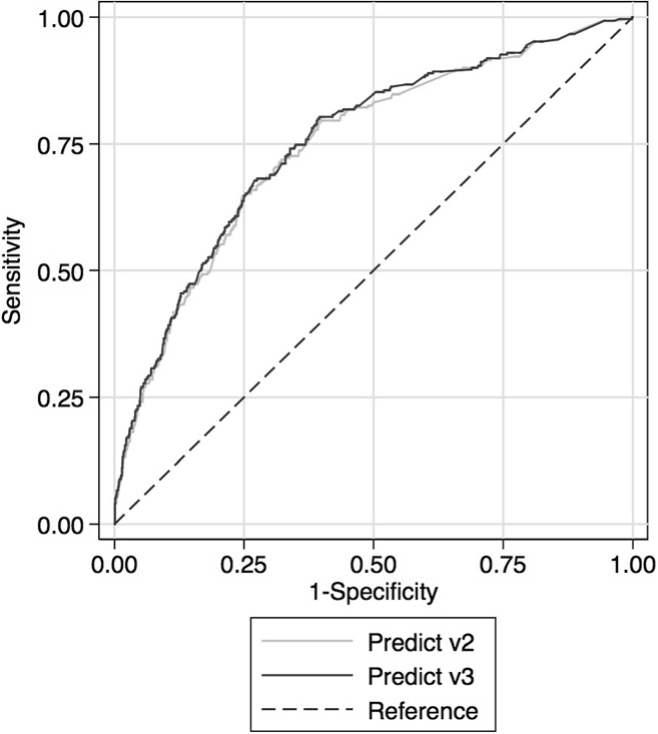
**Receiver operator characteristic curves for breast cancer specific mortality in 1,274 cases with ER positive disease based on PREDICT v2 and PREDICT v3.**

The discrimination of both versions of PREDICT was also good and again was slightly better in v3 than in v2. Discrimination, as estimated from the AUC significantly improved from 0.7611 for v2 to 0.7676 for v3 (p = 0.005). The receiver operating characteristics curves are shown in Figure 2. When all 1,726 patients (ER+ and ER-) were analysed, the addition of KI67 to PREDICT significantly improved the AUC from 0.7546 to 0.7595 (p = 0.0008).

## Discussion and conclusions

Addition of KI67 to the Predict model has significantly improved both calibration and discrimination of PREDICT and this version (v3) of the model is now freely available online at http://www.predict.nhs.uk. It is anticipated that this improvement in model performance will contribute to more accurate predictions of the chemotherapy benefit for individual patients. Both versions of PREDICT, with (v3) and without KI67 (v2), underestimated the number of breast cancer deaths by 8% and 11% respectively in this case cohort. This may be partly explained by the fact that the Nottingham dataset is an older cohort of patients diagnosed from 1989 to 1998, whereas PREDICT is based on women diagnosed in East Anglia, UK from 1999 to 2003.

Several multi-gene expression assays are now available for use in breast cancer management. They are based on mRNA expression in up to 70 cell cycle and proliferation genes [17–19]. The Genomic Health recurrence score (Onco*type* Dx® RS) is a prognosticator (breast cancer recurrence) based on a 21 gene expression profile. Onco*type* Dx® has recently been recommended by NICE (DG10) for use in women with oestrogen receptor positive, lymph node negative and HER2 negative early breast cancer to guide chemotherapy decisions if the person is assessed as being at intermediate risk using routine parameters, and where the information on the biological features of the cancer provided by Oncot*ype* DX® is likely to help in predicting the course of the disease. While the analytic validity of the gene expression component of the Oncot*ype* DX® RS is well established, the clinical validity – i.e the calibration and discrimination of the recurrence predictions of the Oncot*ype* DX® RS - has not been published. Furthermore, the incremental improvement in discrimination for the Onco*type* DX® RS recurrence predictions over the established prognostic factors included in PREDICT is not known. A recent study has reported that the Oncot*ype* DX® RS is an independent prognostic factor in ER-negative, HER2-negative tumours but the improvement in discrimination from the RS compared to clinical variables was less than the improvement obtained from the improvement obtained by IHC4, an immunohistochemistry test that includes KI67 [20]. Another recent study explored the addition of the 70-gene signature (MammaPrint™) to Predict (v2) in 427 patients with early stage breast cancer and found no significant improvement in 5- or 10-year survival predictions [21].

There has been considerable debate about the utility of KI67 IHC in routine clinical practice, partly because the analytic validity of KI67 measurement by IHC is sub-optimal and the optimal threshold for identifying KI67 positive tumours is not known. However, while such considerations are germane to the incorporation on KI67 IHC into a multi-variable risk prediction model, issues around analytic validity are not of primary importance in this study. The KI67 parameter included in the PREDICT model was derived from data from one study – SEARCH. The validation of the PREDICT risk prediction model utilized data from a completely independent case-cohort for which KI67 had been measured in a completely different laboratory. It is thus likely that the standardization of KI67 was sub-optimal. The calibration and discrimination of PREDICT improved despite this limitation. This emphasizes the point that even a marker measured sub-optimally can have clinical validity when that marker is used in the context of risk prediction.

Inclusion of HER2 and KI67 in PREDICT has significantly improved the performance to estimate breast cancer specific mortality. It is likely that the estimated absolute 10-year benefits of adjuvant chemotherapy will be similarly improved. The authors recognise that there may be a better way to dichotomise KI67 positivity, but the 10% cut-off has been shown previously to be optimal [22], and the use of this simple cut off in our study demonstrated the validity of KI67 as a prognostic marker with improved performance of the PREDICT model. This model, based on traditional clinico-pathological factors as well as IHC detection of 3 IHC markers (ER, HER2 & KI67), now provides an ideal platform to test the incremental improvement with the addition of any new prognostic marker or gene expression profile. Inclusion of progesterone receptor (PR) is the only widely used IHC marker not currently included in the PREDICT model and future studies will explore inclusion of PR. The version of PREDICT that includes KI67 is quick to use, free and available for decision making at the clinician desk-top. Oncot*ype* Dx is now widely used in the USA, but the cost has prevented worldwide adoption for risk assessment in patients with early-stage ER-positive breast cancer. We believe that further research should address whether gene-expression profiles such as Onco*type* Dx actually provide any incremental benefit in risk prediction to that currently provided by the most recent version of PREDICT.

Addition of KI67 to PREDICT has led to a statistically significant improvement in the model performance for ER+ patients and will aid clinical decision making in these patients. Further studies should determine whether other markers including gene expression profiling provide additional prognostic information to that provided by PREDICT.
